# Level of Genetic Diversity in European Bumblebees is Not Determined by Local Species Abundance

**DOI:** 10.3389/fgene.2019.01262

**Published:** 2019-12-10

**Authors:** Kevin Maebe, Reet Karise, Ivan Meeus, Marika Mänd, Guy Smagghe

**Affiliations:** ^1^Department of Plants and Crops, Faculty of Bioscience Engineering, Ghent University, Ghent, Belgium; ^2^Institute of Agricultural and Environmental Sciences, University of Life Sciences, Tartu, Estonia

**Keywords:** genetic diversity, bumblebees, local abundance, microsatellite loci, population genetics

## Abstract

Bumblebee species with declining population trends tend to show lower genetic diversity levels than stable species. The observed difference might be explained by abundance differences, with declining bumblebee species having lower genetic diversity levels simply due to their lower local species abundances. However, whether this holds true is not known. Here, we investigated whether bumblebee local abundances determines population genetic diversity levels. Therefore, local species abundances were measured for bumblebee species at four locations in Belgium and two locations in Estonia during bumblebee foraging years 2013–2017. These locations and countries were chosen to ensure the greatest possible variance in both local abundances and population trends for these species. Hence, genetic diversity levels were obtained for seven species by genotyping collected specimens with 16 microsatellites. Our results showed that the observed patterns in genetic diversity did not depend on local species abundance. So, although declining bumblebee species can become locally abundant, they will still show lower genetic diversity levels than stable species. This result implies that measuring bumblebees’ local abundance cannot be used to directly determine the health status of a population. Furthermore, this result has also major impact on future conservation strategies as increasing the genetic diversity levels of declining species will be very difficult, and habitat quality should be high to maintain their populations, otherwise these species are doomed to disappear first.

## Introduction

An essential factor for species survival is the level of genetic diversity present within its populations. Species which have a low level of intraspecific genetic diversity will have more limited potential to adapt to current and future changes in the environment (Frankham, 2005; Zayed, 2009; Habel et al., 2014; Koch et al., 2017). Small populations are more likely to have lower genetic diversity levels than large populations due to the impact of genetic drift, the random loss of genetic diversity over time (Reed and Frankham, 2003; Frankham, 2005; Zayed, 2009). However, high levels of gene flow and improved dispersal capacities can compensate for loss of allelic richness due to drift. In smaller less-connected populations, gene flow may be more limited, decreasing its buffering effect, which leads to lower genetic diversity and increase brother–sister mating chances, and in turn to inbreeding and inbreeding depression (Frankham, 2005; Zayed, 2009; Habel et al., 2014). The latter dynamics can further diminish genetic variability within small populations, potentially creating a vicious circle, known as the extinction vortex, which could ultimately lead to extinction (Frankham, 2005; Zayed, 2009; Habel et al., 2014).

Bumblebees are eusocial insects, living in large colonies mostly founded by one queen and many workers (Alford, 1975; Goulson, 2010). In most bumble bee species, queens are monoandrous with their offspring being fullsibs, having 75% or more genetic similarity between them (Estoup et al., 1995; Schmid-Hempel and Schmid-Hempel, 2000; Goulson, 2010). The high relatedness of sisters can be explained by bumblebees’ haplodiploid sex-determination system in which unfertilized eggs, being hemizygous at the single sex-determining locus, will develop in haploid males or drones, and at which fertilized eggs will develop in diploid female offspring (workers and daughter queens) when being heterozygous at the sex locus. However, fertilized eggs can also develop into diploid males if being homozygous (Duchateau et al., 1994; Cook and Crozier, 1995; Whitehorn et al., 2009). The probability of such matched-pair matings at the sex locus is normally low due to the rather high number of alleles at this loci (46 alleles in *B. terrestris*; Duchateau et al., 1994). However, the presence of diploid drones will cause negative effects on population growth and survival, particularly in populations with low variation at the sex locus, which are typically small and/or inbred populations (Cook and Crozier, 1995; Duchateau and Marien, 1995; Gerloff and Schmid-Hempel, 2005; Whitehorn et al., 2009).

Multiple studies have shown that bumblebee species with declining population trends tend to have lower genetic diversity levels compared with stable bumblebee species (Darvill et al., 2006; Ellis et al., 2006; Goulson et al., 2008; Charman et al., 2010; Cameron et al., 2011). This observation has been often explained as a reduction of genetic diversity over time (Goulson et al., 2008; Charman et al., 2010) due to the impact of one or multiple possible drivers of bee decline, e.g. agricultural intensification, new pathogens, and climate changes, lowering population sizes (Potts et al., 2010; Meeus et al., 2011; Vanbergen et al., 2013; Rasmont et al., 2015). However, studies using historical populations found a similar difference in genetic variation between declining and stable bumblebee species (Lozier and Cameron, 2009; Lozier et al., 2011; Maebe et al., 2015; Maebe et al., 2016), detecting no major drop in genetic diversity over one century in Belgium, possibly due to dispersal countering drift effects (Maebe et al., 2016). The latter result indicates that, at least for Belgian populations, species abundance, and genetic diversity are not linked. This is in conflict with the rather general theory, that when a certain species is locally abundant, its large population should have a higher amount of genetic variation than observed at a location where this species is less abundantly present (Reed and Frankham, 2003; Frankham, 2005; Zayed, 2009). However, for social insects, such as bumblebees, this might thus not be always true.

Here, we investigated if species abundance determines genetic diversity levels in eusocial bumblebee species, and this on both local and larger scale. Hence, we searched whether genetic diversity is linked with species IUCN status (or European pattern of occurrence), and compared this with local abundance measures. Therefore, specimens of seven bumblebee species (*B. ruderarius*, *B. soroeensis*, *B. sylvarum, B. hortorum*, *B. hypnorum*, *B. lapidarius*, and *B. pascuorum*) were collected from two countries: Belgium and Estonia. Three species have restricted distribution and declining population trends in Belgium (*B. ruderarius*, *B. soroeensis*, and *B. sylvarum*), while the remaining four species have a more widespread distribution and showed fairly stable population trends (*B. hortorum*, *B. hypnorum*, *B. lapidarius*, and *B. pascuorum*) (division based on Maebe et al., 2016 and refs herein). Although the latter bumblebee species are also dominantly present in Estonia, these species occur in different abundances, while the "declining" species are more abundantly present and even showing increasing population trends (**Table 1**). Bumblebee workers were collected from six locations, four in Belgium (Francorchamps, Moorsel, Torgny, and Trivières) and two in Estonia (Harjumaa and Põlvamaa) during bumblebee foraging years from 2013 to 2017. For each species, genetic diversity was estimated from each sampling location using 16 microsatellite markers. Furthermore, species local abundance was determined as its relative abundance (= its present abundance compared to the total species abundance presented at that location). This approach allowed us to investigate the link between bumblebee local abundance and genetic diversity.

**Table 1:** Species population trends for Estonian and Belgian bumblebees.

Species	IUCN Red List status	IUCN based group	BELGIUM^1^				ESTONIA^2^			
			Abundance	Abundance	Trend	Status	Abundance	Abundance	Trend	Status
			1910–1930	1990–2016			1955-1967	2009-2018		
*Bombus hortorum*	NT	Stable	9.85%	3.11%	−68.43%	Declining	12.50%	7.13%	−42.99%	Declining
*Bombus pascuorum*	LC	Stable	28.31%	30.48%	+7.66%	Not-declining	10.60%	11.24%	+6.01%	Not-declining
*Bombus ruderarius*	EN	Declining	1.91%	0.30%	−84.29%	Declining	6.10%	6.35%	+4.09%	Not-declining
*Bombus sylvarum*	CE	Declining	1.20%	0.19%	−84.17%	Declining	6.50%	9.18%	+41.26%	Not-declining
*Bombus lapidarius*	LC	Stable	14.13%	17.05%	+20.67%	Not-declining	11.70%	28.80%	+146.19%	Not-declining
*Bombus soroeensis*	VU	Declining	0.62%	0.32%	−48.39%	Declining	0.10%	8.27%	+8171.98%	Not-declining
*Bombus hypnorum*	LC	Stable	1.46%	3.93%	+169.18%	Not-declining	3.40%	1.92%	−43.46%	Declining

Based on the difference in relative abundance between two time periods and each species IUCN red list status (LC, Least Concern; NT, Near Threatened; VU, Vulnerable; EN, Endangered; and CE, Critically Endangered; Rasmont et al., 2015).

^1^Vray et al. (2019).

^2^Kotkas (1968) and Viik (2019).

## Material and Methods

### Sampling and Proportional Abundance Measurement

**Figure 1:**
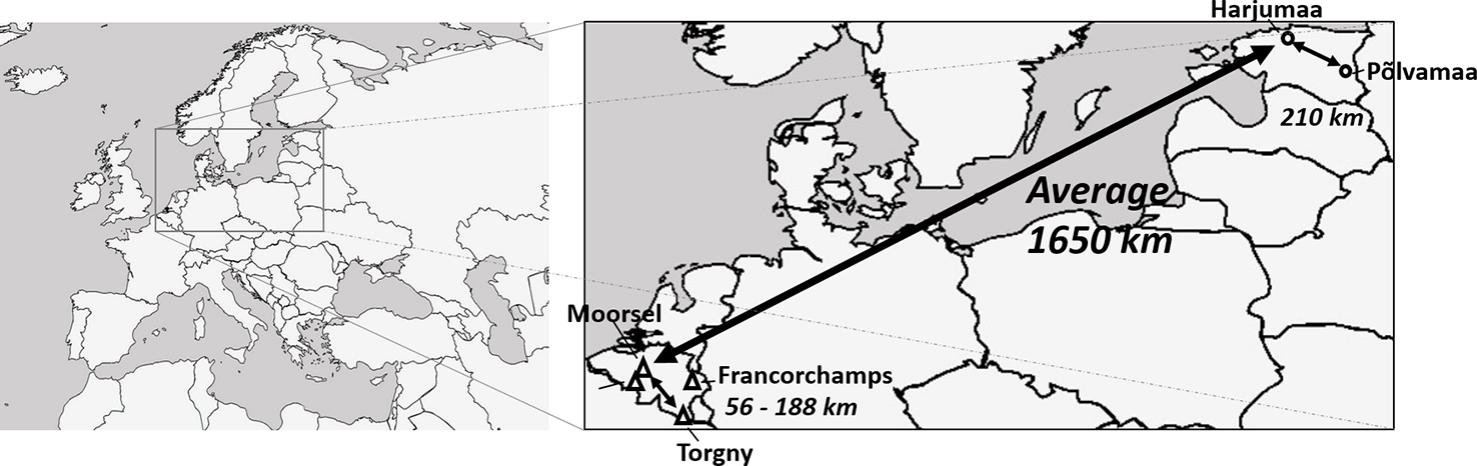
Map of sampling locations in Belgium and Estonia (adapted Figure from Maebe et al. 2019 under Creative Commons Attribution 4.0 International License; https://creativecommons.org/licenses/by/4.0/).

A sampling effort for specimens from seven bumblebee species (*B. hortorum*, *B. hypnorum*, *B. pascuorum, B. ruderarius*, *B. soroeensis*, *B. sylvarum*, and *B. hypnorum*) was performed at four sampling locations in Belgium and two in Estonia during bumblebee foraging seasons from 2013 to 2017. Selected sampling locations within both countries were around 56–210 km apart, while the distance between both countries ranged 1,650 km (**Figure 1**). Specimens were collected from each location for three species, *B. hortorum*, *B. hypnorum*, and *B. pascuorum*. Unfortunately, the other species were only present at three out of seven sampling locations (one Estonian and two Belgian locations for *B. hypnorum*, one Belgian and two Estonian locations for *B. ruderarius*, *B. soroeensis*, and *B. sylvarum*; **Table 2**).

**Table 2:** Estimated mean *H*_E_ and *A*_R_ (± SE) in Belgian and Estonian bumblebee populations.

Species	N	Location	Country	*H*_E_	SE	*A*_R_	SE
*B. hortorum*	25	Harjumaa	Estonia	0.546	0.088	3.01	0.36
*B. hortorum*	37	Põlvamaa	Estonia	0.567	0.093	3.16	0.39
*B. hortorum*	25	Francorchamp	Belgium	0.587	0.092	3.23	0.38
*B. hortorum*	17	Trivières	Belgium	0.570	0.087	3.09	0.35
*B. hortorum*	19	Moorsel	Belgium	0.580	0.095	3.21	0.38
*B. hortorum*	23	Torgny	Belgium	0.550	0.093	3.06	0.38
*B. hypnorum*	3	Põlvamaa	Estonia	0.329	0.090	1.94	0.27
*B. hypnorum*	8	Moorsel	Belgium	0.371	0.071	1.94	0.19
*B. hypnorum*	10	Torgny	Belgium	0.388	0.071	2.03	0.19
*B. lapidarius*	25	Harjumaa	Estonia	0.663	0.064	3.29	0.26
*B. lapidarius*	22	Põlvamaa	Estonia	0.651	0.071	3.25	0.27
*B. lapidarius*	23	Francorchamp	Belgium	0.720	0.056	3.57	0.24
*B. lapidarius*	22	Trivières	Belgium	0.742	0.047	3.65	0.22
*B. lapidarius*	19	Moorsel	Belgium	0.719	0.054	3.57	0.24
*B. lapidarius*	21	Torgny	Belgium	0.739	0.054	3.66	0.25
*B. pascuorum*	24	Harjumaa	Estonia	0.445	0.087	2.53	0.32
*B. pascuorum*	33	Põlvamaa	Estonia	0.443	0.084	2.50	0.32
*B. pascuorum*	26	Francorchamp	Belgium	0.456	0.085	2.56	0.32
*B. pascuorum*	23	Trivières	Belgium	0.436	0.083	2.46	0.31
*B. pascuorum*	21	Moorsel	Belgium	0.443	0.087	2.51	0.33
*B. pascuorum*	20	Torgny	Belgium	0.398	0.085	2.34	0.31
*B. ruderarius*	8	Harjumaa	Estonia	0.366	0.097	2.27	0.35
*B. ruderarius*	13	Põlvamaa	Estonia	0.313	0.094	2.05	0.33
*B. ruderarius*	10	Torgny	Belgium	0.321	0.102	2.16	0.37
*B. soroeensis*	23	Harjumaa	Estonia	0.493	0.100	2.84	0.41
*B. soroeensis*	22	Põlvamaa	Estonia	0.486	0.098	2.85	0.41
*B. soroeensis*	9	Torgny	Belgium	0.430	0.095	2.49	0.35
*B. sylvarum*	47	Harjumaa	Estonia	0.269	0.080	1.87	0.28
*B. sylvarum*	37	Põlvamaa	Estonia	0.288	0.084	1.93	0.31
*B. sylvarum*	13	Torgny	Belgium	0.330	0.084	2.07	0.31

392 bumblebee specimens were collected from the four selected Belgian locations: Francorchamps, Moorsel, Torgny, and Trivières, and were already genotyped at 16 microsatellite loci within a previous study (Maebe et al., 2016; **Table 2**). These specimens were part of an intensive sampling performed within a national bumblebee project (Belbees) during 3 days in the bumblebee foraging season of 2013 and 5 days in 2015. At pollinator-suitable weather conditions, all encountered bumblebees were sampled during straight ahead transect walks using a net. Species abundances were calculated as the proportion of a species abundance versus the total bumblebee abundance collected at that particular location (**Table 3**).

**Table 3:** Bumblebee relative abundances in Belgium and Estonia locations.

Species	Abundancy (proportion in %)					
	BELGIUM^1^				ESTONIA^2^	
	Torgny	Moorsel	Francorchamps	Trivières	Põlvamaa	Harjumaa
*B. barbutellus*	0.69	0.00	0.00	0.10	0.0	0.0
*B. bohemicus*	1.57	0.00	2.20	0.00	0.0	0.0
*B. campestris*	1.08	0.13	0.37	0.19	0.0	0.0
*B. cryptarum*	0.10	0.00	4.22	0.00	0.0	0.0
*B. distinguendus*	0.00	0.00	0.00	0.00	1.2	0.7
*B. hortorum*	3.74	4.15	4.31	2.30	8.6	3.3
*B. humilis*	1.67	0.00	0.00	0.00	1.3	0.0
*B. hypnorum*	2.17	5.40	1.47	2.49	1.6	0.3
*B. jonellus*	0.00	0.00	0.00	0.00	0.6	0.0
*B. lapidarius*	26.48	10.30	6.97	17.82	15.7	53.1
*B. lucorum*	3.05	0.63	20.71	0.77	18.5	11.8
*B. muscorum*	0.00	0.00	0.00	0.00	0.7	0.0
*B. pascuorum*	19.19	60.68	44.36	46.55	16.9	15.8
*B. pratorum*	2.95	8.79	9.17	3.74	2.9	0.6
*B. ruderarius*	2.85	0.00	0.00	0.00	7.4	1.5
*B. rupestris*	4.63	0.00	0.09	0.00	0.0	0.0
*B. schrencki*	0.00	0.00	0.00	0.00	1.5	0.3
*B. soroeensis*	3.05	0.00	0.09	0.00	6.9	8.6
*B. subterraneus*	0.00	0.00	0.00	0.00	2.7	0.0
*B. sylvarum*	4.82	0.00	0.00	0.00	6.8	1.3
*B. sylvestris*	1.57	0.00	0.73	0.19	0.0	0.0
*B. terrestris*	17.03	9.92	5.13	25.67	1.7	0.5
*B. vestalis*	3.35	0.00	0.18	0.19	0.0	0.0
*B. veteranus*	0.00	0.00	0.00	0.00	5.0	2.3
***Total***	***100***	***100***	***100***	***100***	***100***	***100***

^1^Vray et al. (2019).

^2^Marja et al. (2014).

In Estonia, Harjumaa and Põlvamaa were selected as sampling locations (**Table 2**). Sampling was performed during the bumblebee foraging seasons of 2015 and 2017. Individual specimens were collected with small glass jars from a flower, killed with chloroform, and stored in a −20°C freezer. The Estonian bumblebee abundance data were derived from Marja et al. (2014), from which the North and South Estonia abundances were used as estimates for Harjumaa and Põlvamaa, respectively. Here, per species local abundances were also calculated (**Table 3**).

### DNA Extraction and Microsatellite Protocol

DNA extraction and analysis were performed only on the Estonian specimens, with the same method as was done in a previous study for the Belgian specimens (see Maebe et al., 2016). One middle leg of an individual bumblebee worker was used for DNA extraction using a Chelex DNA extraction protocol (see methods described in Maebe et al., 2015). Specimens were genotyped with 16 microsatellite (MS) loci which gave reliable signals in previous research using different bumblebee species (**Supplementary Table 1**; Maebe et al., 2015; Maebe et al., 2016; Maebe et al., 2018). MS were amplified using the Type-it QIAGEN PCR kit using four multiplex mixes as described in Maebe et al. (2016). The PCR protocol, capillary electrophoreses, and fragment scoring were performed with the method as described in Maebe et al. (2013).

### Linkage Disequilibrium, Hardy–Weinberg Equilibrium, and Sister Detection

Linkage disequilibrium, Hardy–Weinberg equilibrium (HW) deviations, and the presence of null alleles were tested for all populations by using Fstat 2.9.3 (Goudet, 2001), GenAlEx v6.5 (Peakall and Smouse, 2006), and Microchecker (Van Oosterhout, 2004), respectively. All specimens which could not be scored in a reliable manner for 10 or more loci were removed. Furthermore, after detection of full-siblings with Colony 2.0 (Wang, 2004) employing corrections for genotyping errors (5% per locus) and by the two allele algorithm and consensus method implemented in Kinalyzer (Ashley et al., 2009), we retained only one sister per colony (see also Maebe et al., 2015).

### Genetic Diversity

GenAlEx v6.5 (Peakall and Smouse, 2006) was used to estimate Nei’s unbiased expected heterozygosity (*H*_E_) and observed heterozygosity (*H*_O_) (Nei, 1978) per population and for all species. With Hp-Rare 1.1 (Kalinowski, 2015) we estimated sample size-corrected private allelic richness (*A*_R_) normalized to 10 gene copies.

### Impact of Local Abundance on Level of Genetic Diversity

To be able to detect if species abundance influences the observed differences in species genetic diversity, Linear Mixed Models (LMMs) were performed for each species and both genetic diversity parameters (*A*_R_ and *H*_E_) in RStudio (R Development Core Team, 2008) with R package lme4 version 1.1-10 (Bates et al., 2015) as described in Maebe et al., 2018. In short: species, local abundance, and location were set as fixed factors and microsatellite loci as a random factor. The best model fitting our data was selected based on the Akaike’s Information Criterion (AIC) by using the “dredge” command within the MUMIn package (Barton 2015; Maebe et al., 2016; Maebe et al., 2018). The main effect of the factor of interest was analyzed for each selected LMM by performing Likelihood Ratio Tests (LTR) in which the model with factors was compared with a “null” model without these factors (Maebe et al., 2018). After computing the marginal and conditional coefficient of determination, Tukey HSD *post hoc* comparisons were performed using the R package multcomp to find differences in genetic diversity due to species local abundance, location and/or species (Hothorn et al., 2008; described in Maebe et al., 2018, and references therein).

### Genetic Diversity and IUCN Status

Based on the IUCN bumblebee red list status, bumblebee species were organized in two groups, either “stable” or “declining” species (**Table 1**). The group of the “stable” species consisted out of four species (*B. hortorum*, *B. hypnorum*, *B. lapidarius*, and *B. pascuorum*) of which all species had an IUCN status of “Least Concern” (LC), except for *B. hortorum* which have a “Near Threatened” (NT) status (Rasmont et al., 2015). The three remaining species, *B. ruderarius, B. soroeensis*, and *B. sylvarum*, were grouped as “declining” species, as they all have a threatened IUCN status ("Endangered” (EN), “Vulnerable” (VU), and “Critically Endangered” (CE), respectively; Rasmont et al., 2015; **Table 1**). Secondly, for each country, bumblebee species were divided in two groups based on their population trends, being “declining” or “not-declining” species. Such a population trend was made for each species based on the difference in relative abundance of that species between two time periods: for Belgium between 1910–1930 and 1990–2016 (Vray et al., 2019; **Table 3**), while for Estonia between 1955–1967 and 2009–2018 (Kotkas, 1968; Viik, 2019; **Table 3**). Genetic diversity parameters (*A*_R_ and *H*_E_) were compared between the different groups by LMMs in RStudio (R Development Core Team, 2008) with R package lme4 version 1.1-10 (Bates et al., 2015). Here, models were run with microsatellite “loci” as random factor, and “group” as fixed factor. Tukey *post hoc* tests were performed as described above.

## Results

All 16 microsatellites could be amplified and scored reliably in the seven *Bombus* species. In total, 628 specimens (309 Belgian and 319 Estonian specimens) remained for further genetic analysis out of 729 specimens, due to the removal of full-sibs which were detected by Colony 2.0 and Kinalyzer analyses, and discarding of specimens which have more than five loci of missing data from both Belgian and Estonian dataset. Furthermore, no significant linkage disequilibrium between loci were detected, and Hardy–Weinberg equilibrium tests displayed no or only limited heterozygote deficits.

### Correlation Between Local Abundance and Genetic Diversity

**Figure 2:**
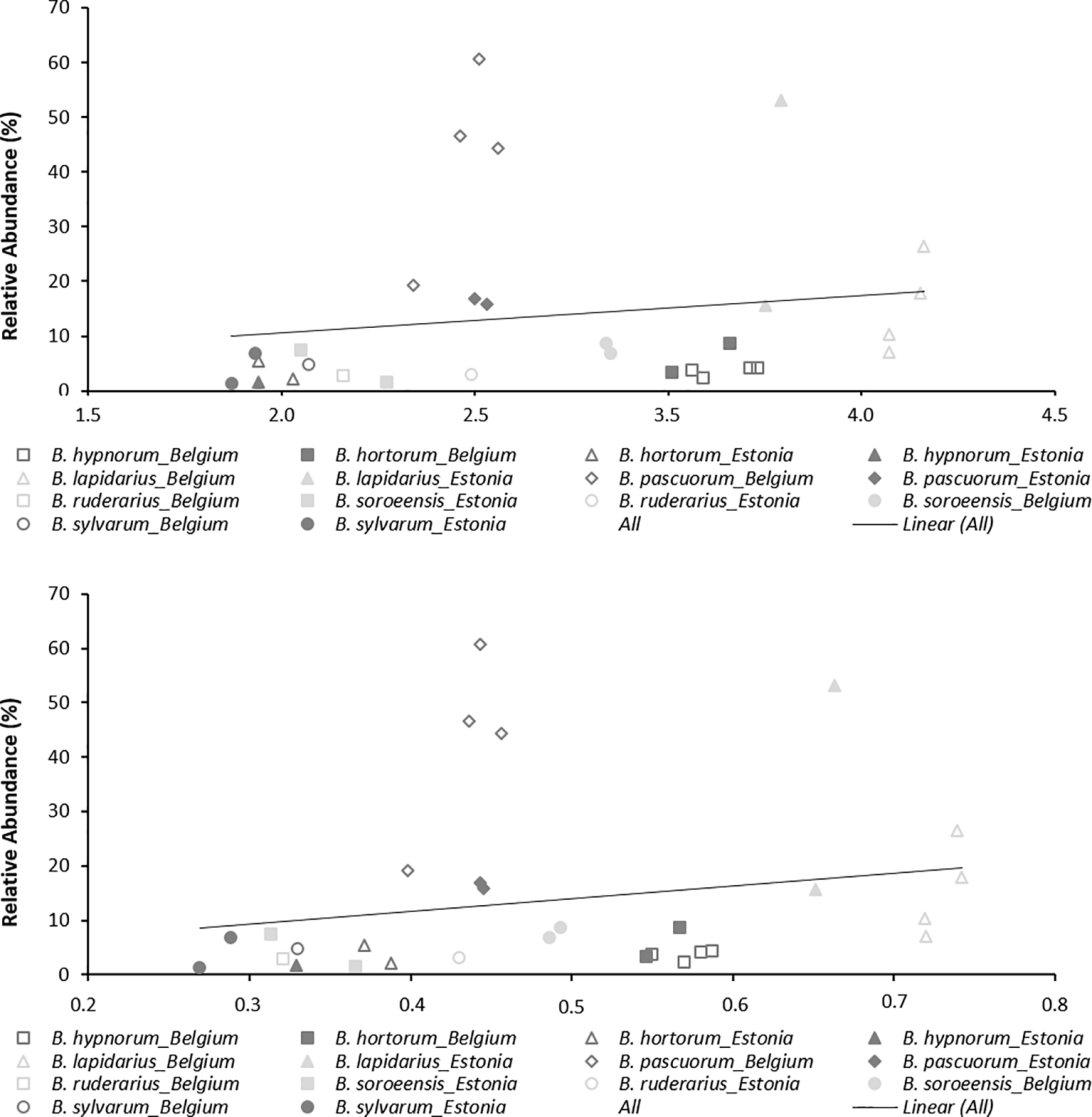
Correlation between relative abundance and genetic diversity parameters (*A*_R_ and *H*_E_).

Overall for populations and species, allelic richness (*A*_R_) ranged from 1.87 to 3.66, with a mean *A*_R_ of 2.70. Mean *H*_E_ was 0.488, with individual population values ranging from 0.269 to 0.739 (**Table 2**). Linear regression showed low and non-significant correlations between local abundance and *A*_R_ or *H*_E_ (R^2^ = 0.0261, p = 0.393; R^2^ = 0.0419, p = 0.278; respectively; **Figure 2**) suggesting that only a low amount of variation of genetic diversity is explained by local abundance. By comparing the AIC scores of the different LMM models, the best model explaining the observed patterns of both genetic diversity parameters was not the model with “abundance”, but with “species” as the only fixed factor (**Table 4**). The importance of the factor “species” for both *A*_R_ and *H*_E_ was clearly shown by comparing the models with and without “species” as fixed factor (LRT, χ^2^ = 154.07, d.f. = 6, *p* < 0.001; and χ^2^ = 140.72, d.f. = 6, *p* < 0.001, respectively). However, addition of abundance as an extra fixed factor had no significant impact on the model, as became clear after comparing the models with both “species” and “abundance” as fixed factors to the models without abundance as additional fixed factor (LRT, *A*_R_ and *H*_E_: χ^2^ = 0.1307, d.f. = 1 *p* = 0.718; and χ^2^ = 0.0598, d.f. = 1, *p* = 0.807, respectively). Marginal and conditional R^2^ were similar for both *A*_R_ and *H*_E_, 16.4% and 57.1% *versus* 15.2% and 55.7%, respectively (Nakagawa and Schielzeth, 2013). *Post hoc* tests were performed and showed significant differences in genetic diversity levels between the seven bumblebee species (**Table 5** and **Supplementary Tables 2** and **3**).

**Table 4:** Selection of best fitting model explaining the genetic diversity pattern.

A	*A*_R_	Intercept	Abn.	Loc.	Spss	Abn : Loc	Loc : Per	Abn : Per	Abn : Loc:Per	df	logLik	AIC	Delta	Weight
	**M5**	**3.128**	***NA***	***NA***	+	***NA***	***NA***	***NA***	***NA***	**9**	**−666.590**	**1,351.6**	**0.00**	**0.717**
	M6	3.134	+	*NA*	+	*NA*	*NA*	*NA*	*NA*	10	−666.525	1,353.5	1.96	0.270
	M7	3.229	*NA*	+	+	*NA*	*NA*	*NA*	*NA*	14	−665.868	1,360.6	9.08	0.008
**B**	***H*_E_**	**Intercept**	**Abn**.	**Loc**.	**Spss**	**Abn : Loc**	**Loc : Per**	**Abn : Per**	**Abn : Loc:Per**	**df**	**logLik**	**AIC**	**Delta**	**Weight**
	**M5**	**0.567**	***NA***	***NA***	+	***NA***	***NA***	***NA***	***NA***	**9**	**−12.202**	**42.8**	**0.00**	**0.726**
	M6	0.568	+	*NA*	+	*NA*	*NA*	*NA*	*NA*	10	−12.172	44.8	2.03	0.263
	M7	0.584	*NA*	+	+	*NA*	*NA*	*NA*	*NA*	14	−11.525	52.0	9.17	0.007

Of all possible models run under MUMIn using location (= Loc.), species abundance (= Abn), species (= Spss), and time periods (= Per.) as fixed effects, and locus as a random effect, the best fitting linear mixed-effect models (with a delta <10) are given. Based on their high (negative or positive) Akaike’s Information Criterion (AIC), the best models for A_R_ (A) and H_E_ (B) with + = parameters included in the model, and NA, not included parameters. Selected best models for AR and HE are given in bold.

**Table 5:** *Post hoc* results of the selected linear mixed models (LMM).

*A*_R_	*B. hortorum*	*B. hypnorum*	*B. lapidarius*	*B. pascuorum*	*B. ruderarius*	*B. soroeensis*	*B. sylvarum*
*B. hortorum*	−	−	**+**	**−**	**−**	**−**	**−**
*B. hypnorum*	***	−	**+**	**+**	=	**+**	=
*B. lapidarius*	**	***	−	**−**	**−**	**−**	**−**
*B. pascuorum*	***	**	***	−	**−**	=	**−**
*B. ruderarius*	***	NS	***	*	−	**+**	=
*B. soroeensis*	*	***	***	NS	***	−	**−**
*B. sylvarum*	***	NS	***	***	NS	***	−
***H*_E_**	***B. hortorum***	***B. hypnorum***	***B. lapidarius***	***B. pascuorum***	***B. ruderarius***	***B. soroeensis***	***B. sylvarum***
*B. hortorum*	−	**−**	**+**	**−**	**−**	=	**−**
*B. hypnorum*	***	−	**+**	=	=	**+**	=
*B. lapidarius*	***	***	−	**−**	**−**	**−**	**−**
*B. pascuorum*	***	NS	***	−	**−**	=	**−**
*B. ruderarius*	***	NS	***	**	−	**+**	=
*B. soroeensis*	NS	**	***	NS	***	−	**−**
*B. sylvarum*	***	NS	***	***	NS	***	−

Impact of the factor lrdquo;species” in the model on A_R_ and H_E_. Significant levels p < 0.001 = ***, p < 0.01 = **, p < 0.05 = *, and NS = not significant, with in bold and italic negative and positive interactions, respectively.

### Genetic Diversity Differences Between Species Groups?

Comparing both *A*_R_ and *H*_E_ between the two IUCN groups showed a significantly higher genetic diversity level in “stable" bumblebee species compared to the contemporary “declining" species (*post hoc* tests: *A*_R_, *t* = 5.874, *p* < 0.001, and *H*_E_, *t* = 6.226, *p* < 0.001; **Table 6**). However, genetic diversity was similar when comparing populations with "not-declining" compared with those with "declining" population trends (*post hoc* tests; *A*_R_, *t* = −0.489, *p* = 0.625, and *H*_E_, *t* = 0.172, *p* = 0.864; **Table 6**).

**Table 6:** *Post hoc* results of the LMM comparing genetic diversity between species groups LMM’s results for (A) *A*_R_ and (B) *H*_E_.

A.	*A*_R_	Estimate	SE	*t*-value	*p*-value	Sign. level
	IUCN_Threatened *vs* Non-threatened	0.611	0.104	5.874	**<0.001**	***
	Declining *vs* Non-declining	−0.051	0.105	−0.489	0.625	
**B**.	***H*_E_**	**Estimate**	**SE**	***t*-value**	***p*-value**	**Sign. Level**
	IUCN_Threatened *vs* Non-threatened	0.373	0.060	6.226	**<0.001**	***
	Declining *vs* Non-declining	0.005	0.026	0.172	0.864	

With the estimate, standard error (SE) and p-value of each factor in the model. Significant factors are indicated in bold, with significant levels p < 0.001 = ***, p < 0.01 = **, and p < 0.05 = *.

## Discussion

In this study, we investigated whether genetic diversity is determined by species local abundance. By genotyping specimens of seven bumblebee species from Belgium and Estonia with 16 microsatellites, we were able to estimate genetic diversity and to compare this with local species abundance. This approach allowed us to conclude that observed genetic diversity patterns were not significantly dependent on species local abundance. Indeed, the best models explaining genetic diversity patterns did not include local abundance as a factor, and both *A*_R_ and *H*_E_ were not significantly correlated with abundance (**Figure 2**). This is rather unexpected as one might think that when a certain species is locally abundant, this large population should normally have a higher amount of genetic variation than may be observed at a location where this species is less abundantly present. However, this is not always true. Indeed, when the local environment has enough food, resources, and is well suited to harbor a large population of bumblebees, then a bumblebee population can become locally large without increasing its genetic diversity levels. This may be partly explained by bumblebees’ haplodiploid sex-determination system in which unfertilized eggs develop into haploid males and fertilized eggs into diploid females, where workers do not mate and normally do not contribute to the next generation, and where each colony consists of a single mated queen and her offspring (Alford, 1975; Goulson, 2010). Such colonies can have large numbers of specimens which are in fact all "clones" with 75% or more genetic similarity between them (Goulson, 2010). A population consisting of large colonies founded by closely related queens will harbor many closely related workers and thus can have large population numbers but lower genetic variation than one might expect. Furthermore, several studies have shown that bumblebees are able to recover from genetic bottlenecks by increasing population sizes without increasing their genetic diversity levels (Schmid-Hempel et al., 2007). Although these populations are characterized with having many specimens, and thus are at first glance in a healthy condition, these populations are under severe fitness risks, due to the low genetic variation which makes these populations vulnerable to additional future environmental changes (Frankham, 2005; Zayed, 2009; Habel et al., 2014; Koch et al., 2017). Indeed, populations with low genetic variation show less population growth and survival (Whitehorn et al., 2009), and are more susceptible to diseases (Whitehorn et al., 2011; Cameron et al., 2011; Whitehorn et al., 2014). Hence, when neighboring populations have rather similar levels of genetic diversity, then increasing a populations’ genetic diversity through high amounts of gene flow will not be possible within a short time span, making these populations even at higher risk. The latter, implying that neighboring populations have similar genetic diversity cannot be proven in our study, as no in depth sampling of multiple locations was performed and much variation may thus be missed from unsampled populations. However, the similar genetic diversity levels we observed within the populations of a certain bumblebee species (**Table 2**), suggest that genetic diversity levels in neighboring populations may be very similar. This in turn would mean that for conservational purposes, increasing genetic diversity levels may not so easily be achieved.

Here, grouping the bumblebee species by IUCN status into “declining” and “stable” (**Table 6**) showed similar results as described by Maebe et al. (2016), confirming lower genetic diversity levels within the populations of declining species than observed within widespread, stable species (as been observed and/or discussed in: Darvill et al., 2006; Ellis et al., 2006; Goulson et al., 2008; Charman et al., 2010; Cameron et al., 2011). Furthermore, our results showed clear differentiation of genetic diversity based on species. Indeed, the best models explaining observed *A*_R_ and *H*_E_ included species as fixed factor (**Table 4**), and *post hoc* tests showed multiple significant differences in genetic diversity between species (**Table 5**). Those results assume that the maximum level of genetic diversity which a species could achieve might be always lower than in other species. The latter might be due to species-specific population characteristics such as lower queen production, smaller colonies, low dispersal capacity, etc., which all could limit the generation of higher genetic diversity levels within the populations of a certain species, and/or due to past population dynamics which have altered diversity levels within species’ populations in the past (as hypothesized in Maebe et al., 2016). The hypothesis of past population dynamics influencing genetic variability has been raised attention as a recent study showed a clear drop in genetic diversity over time in south-Brazilian bumblebee species (Maebe et al., 2018) which contradicted the temporal stability observed within the European bumblebees (Maebe et al., 2016). The authors explained this difference due to deforestation differences between both continents, hypothesizing that in Europe major reductions in genetic diversity occurred earlier in time (Maebe et al., 2018). Whether this holds true must be further investigated, but the lower levels in genetic diversity observed in certain species might thus be due to different impact of land-use changes, such as deforestation, on particular bumblebee species.

To conclude, our results showed that measuring bumblebees’ local abundance cannot be used to directly determine the health status of a population, especially on a longer term. Furthermore, as bumblebee species with less genetic diversity have lower fitness (e.g. Cameron et al., 2011; Whitehorn et al., 2011; Whitehorn et al., 2014), it seems that for conservation purposes, measuring genetic diversity parameters can give a good prediction value whether a certain population or bumblebee species is more or less vulnerable towards future population decline.

## Data Availability Statement

Microsatellite genotypes of each specimen are archived at DRYAD: https://doi.org/10.5061/dryad.7q3n40d.

## Author Contributions

KM and IM designed the research. RK provided specimens. KM analyzed the data. KM wrote the paper, while all co-authors (RK, IM, MM, and GS) helped in improving the manuscript.

## Funding

This study was funded by the Belgian Science Policy (BELSPO) as part of the Belbees project (grant BR/132/A1/BELBEES), the Research Foundation-Flanders (FWO) under EOS Project named CLIPS (Grant 3094785) and research project (Grant 3G042618), and the Institutional Research Funding (IUT36-2) of the Estonian Ministry of Education; European Regional Development Fund project ForBee of RITA program.

## Conflict of Interest

The authors declare that the research was conducted in the absence of any commercial or financial relationships that could be construed as a potential conflict of interest.
