# Supplementary material for: Level of Genetic Diversity in European Bumblebees is Not Determined by Local Species Abundance
Source: Front Genet. 2019 Dec 10;10:1262. doi: 10.3389/fgene.2019.01262 (PMC6914865; doi:10.3389/fgene.2019.01262)
Supplement: Supplementary file 1 [file DataSheet_1.docx]

**Level of Genetic Diversity in European Bumblebees is Not Determined by Local Species Abundance**

Kevin Maebe^1^, Reet Karise^2^, Ivan Meeus^1^, Marika Mänd^2^_,_ and Guy Smagghe^1*^

^1^Department Plants and Crops, Faculty of Bioscience Engineering, Ghent University, Coupure links 653, B-9000, Ghent, Belgium

^2^Institute of Agricultural and Environmental Sciences, University of Life Sciences, Tartu, Estonia

* Corresponding author: prof. Guy Smagghe, [guy.smagghe@ugent.be](mailto:guy.smagghe@ugent.be)

**Supplementary Table 1. List of each microsatellite primer and multiplex used**. Forward and reverse primer sequences, fluorescent dyes, multiplex (MP), annealing temperature (*Ta*), original size range of the PCR product, location in *B. terrestris* genome (LG) and the original reference are given for each SSR marker.

| **Marker** | **MP** | **Dye** | **LG *B.t.*** | **Primer Sequence Forward** | **Primer Sequence Reverse** | ***Ta*** | **Original Range** | **Origin** |
| --- | --- | --- | --- | --- | --- | --- | --- | --- |
| BL13 | 1 | PET | B15 | CGAATGTTGGGATTTTCGTG | GCGAGTACGTGTACGTGTTCTATG | 53 | 205-217 | Reber-Funk et al., 2006 |
| BT02 | 1 | NED | B11 | AGGAACCGAGCGATAGAACCAC | GCTTTGCCTTTCCATCTTGCTG | 53 | 175-183 | Reber-Funk et al., 2006 |
| BT23 | 1 | FAM | B11 | GCAACAGAAAATCGTCGGTAGTG | GCGGCAATAAAGCAATCGG | 54 | 198-216 | Reber-Funk et al., 2006 |
| BT24 | 1 | VIC | B07 | TCTTTCCGTTTTCCCCCTG | CACCCACTTACATACATACACGCTC | 52 | 227-257 | Reber-Funk et al., 2006 |
| BL02 | 2 | NED | B01 | GAACAGTGAGAGCGAGGAACAGAG | TTGCCACGTATATCCGAGCGAACC | 52 | 163-171 | Reber-Funk et al., 2006 |
| BT04 | 2 | FAM | B13 | GAGAGAGATCGAATGGTGAGAGC | TGAGCACGTTCTTTCGTTCAC | 52 | 183-199 | Reber-Funk et al., 2006 |
| BT08 | 2 | PET | B03 | AGAACCTCCGTATCCCTTCG | AGCCTACCCAGTGCTGAAAC | 52 | 208-230 | Reber-Funk et al., 2006 |
| BT10 | 2 | VIC | B08 | TCTTGCTATCCACCACCCGC | GGACAGAAGCATAGACGCACCG | 53 | 178-188 | Reber-Funk et al., 2006 |
| B100 | 3 | FAM | B03 | CGTCCTCGTATCGGGCTAAC | CGTGGAAACGTCGTGACG | 58 | 146-198 | Estoup et al., 1995;1996 |
| B11 | 3 | NED |  | GCAACGAAACTCGAAATCG | GTTCATCCAAGTTTCATCCG | 52 | 124-136 | Estoup et al., 1995;1996 |
| B126 | 3 | PET | B08 | GCTTGCTGGTGAATTGTGC | CGATTCTCTCGTGTACTCC | 56 | 172-176 | Estoup et al., 1995;1996 |
| B132 | 3 | VIC | B11 | GAAATTCGTGCGGAGGG | CAGAGAACTACCTAGTGCTACGC | 58 | 148-213 | Estoup et al., 1995;1996 |
| 0294_10o4 | 4 | FAM | B09 | AGTACGATAAAGCCAGGAAAG | TGTATGCCTATTGTACGAGTGT | 55 | 169-177 | Stolle et al., 2011 |
| 0304_9i13 | 4 | NED | B04 | GTATGAGTGAGTGATGTGCAAG | CCCTTCATCTCTGAACAATATC | 55 | 154-160 | Stolle et al., 2011 |
| 0810_65a23 | 4 | PET | B06 | TTAACAAATCCGAATTTAAAGG | GATAGTGGTTGCTTGTCATCTT | 55 | 136-140 | Stolle et al., 2011 |
| BT05 | 4 | VIC | B02 | TTTCCTATGCCGAACGTCACC | CCCAGATAAAAGACCGCCTCTAGTC | 53 | 194-220 | Reber-Funk et al., 2006 |

**Supplementary Table 2. Post hoc results of the selected linear mixed models (LMM)**. Impact of the factor ‘species’ in the model on *A*_R_. With the estimate, standard error (SE) and *p*-value of the factor in the model. Significant factors are indicated in bold, with significant levels *p* < 0.001 = ***, *p* < 0.01 = ** and *p* < 0.05 = *.

| ***A*_R_** | **Estimate** | **SE** | ***z*-value** | ***p*-value** | **Sign. Level** |
| --- | --- | --- | --- | --- | --- |
| Abundance | -0.001 | 0.004 | -0.362 | 0.718 |  |
| *B. hortorum* vs *B. hypnorum* | -1.158 | 0.162 | -7.144 | **<0.001** | *** |
| *B. hortorum* vs *B. lapidarius* | 0.396 | 0.132 | 2.992 | **0.003** | ** |
| *B. hortorum* vs *B. pascuorum* | -0.645 | 0.132 | -4.884 | **<0.001** | *** |
| *B. hortorum* vs *B. ruderarius* | -0.969 | 0.162 | -5.979 | **<0.001** | *** |
| *B. hortorum* vs *B. soroeensis* | -0.402 | 0.162 | -2.478 | **0.013** | * |
| *B. hortorum* vs *B. sylvarum* | -1.173 | 0.162 | -7.237 | **<0.001** | *** |
| *B. hypnorum* vs *B. lapidarius* | 1.554 | 0.163 | 9.519 | **<0.001** | *** |
| *B. hypnorum* vs *B. pascuorum* | 0.512 | 0.163 | 3.134 | **0.002** | ** |
| *B. hypnorum* vs *B. ruderarius* | 0.189 | 0.189 | 1.002 | 0.316 |  |
| *B. hypnorum* vs *B. soroeensis* | 0.756 | 0.189 | 4.012 | **<0.001** | *** |
| *B. hypnorum* vs *B. sylvarum* | -0.015 | 0.189 | -0.080 | 0.937 |  |
| *B. lapidarius* vs *B. pascuorum* | -1.043 | 0.133 | -7.813 | **<0.001** | *** |
| *B. lapidarius* vs *B. ruderarius* | -1.365 | 0.163 | -8.354 | **<0.001** | *** |
| *B. lapidarius* vs *B. soroeensis* | -0.798 | 0.163 | -4.882 | **<0.001** | *** |
| *B. lapidarius* vs *B. sylvarum* | -1.569 | 0.163 | -9.602 | **<0.001** | *** |
| *B. pascuorum* vs *B. ruderarius* | -0.323 | 0.144 | -2.248 | **0.025** | * |
| *B. pascuorum* vs *B. soroeensis* | 0.245 | 0.144 | 1.704 | 0.088 |  |
| *B. pascuorum* vs *B. sylvarum* | -0.527 | 0.144 | -3.668 | **<0.001** | *** |
| *B. ruderarius* vs *B. soroeensis* | 0.568 | 0.155 | 3.670 | **<0.001** | *** |
| *B. ruderarius* vs *B. sylvarum* | -0.204 | 0.155 | -1.319 | 0.187 |  |
| *B. soroeensis* vs *B. sylvarum* | -0.771 | 0.155 | -4.978 | **<0.001** | *** |

**Supplementary Table 3. Post hoc results of the selected linear mixed models (LMM)**. Impact of the factor ‘species’ in the model on *H*_E_. With the estimate, standard error (SE) and *p*-value of the factor in the model. Significant factors are indicated in bold, with significant levels *p* < 0.001 = ***, *p* < 0.01 = ** and *p* < 0.05 = *.

| ***H*_E_** | **Estimate** | **SE** | ***z*-value** | ***p*-value** | **Sign. level** |
| --- | --- | --- | --- | --- | --- |
| Abundance | -0.0002 | 0.001 | -0.245 | 0.807 |  |
| *B. hortorum* vs *B. hypnorum* | -0.204 | 0.041 | -4.917 | **<0.001** | *** |
| *B. hortorum* vs *B. lapidarius* | 0.139 | 0.034 | 4.103 | **<0.001** | *** |
| *B. hortorum* vs *B. pascuorum* | -0.129 | 0.034 | -3.798 | **<0.001** | *** |
| *B. hortorum* vs *B. ruderarius* | -0.233 | 0.041 | -5.625 | **<0.001** | *** |
| *B. hortorum* vs *B. soroeensis* | -0.076 | 0.041 | -1.840 | 0.066 |  |
| *B. hortorum* vs *B. sylvarum* | -0.271 | 0.041 | -6.527 | **<0.001** | *** |
| *B. hypnorum* vs *B. lapidarius* | 0.343 | 0.042 | 8.201 | **<0.001** | *** |
| *B. hypnorum* vs *B. pascuorum* | 0.075 | 0.042 | 1.801 | 0.072 |  |
| *B. hypnorum* vs *B. ruderarius* | -0.029 | 0.048 | -0.609 | 0.543 |  |
| *B. hypnorum* vs *B. soroeensis* | 0.128 | 0.048 | 2.644 | **0.008** | ** |
| *B. hypnorum* vs *B. sylvarum* | -0.067 | 0.048 | -1.384 | 0.166 |  |
| *B. lapidarius* vs *B. pascuorum* | -0.268 | 0.033 | -8.099 | **<0.001** | *** |
| *B. lapidarius* vs *B. ruderarius* | -0.372 | 0.040 | -9.201 | **<0.001** | *** |
| *B. lapidarius* vs *B. soroeensis* | -0.215 | 0.040 | -5.320 | **<0.001** | *** |
| *B. lapidarius* vs *B. sylvarum* | -0.410 | 0.040 | -10.125 | **<0.001** | *** |
| *B. pascuorum* vs *B. ruderarius* | -0.105 | 0.038 | -2.778 | **0.005** | ** |
| *B. pascuorum* vs *B. soroeensis* | 0.052 | 0.038 | 1.388 | 0.165 |  |
| *B. pascuorum* vs *B. sylvarum* | -0.142 | 0.038 | -3.771 | **<0.001** | *** |
| *B. ruderarius* vs *B. soroeensis* | 0.157 | 0.040 | 3.905 | **<0.001** | *** |
| *B. ruderarius* vs *B. sylvarum* | -0.037 | 0.040 | -0.931 | 0.352 |  |
| *B. soroeensis* vs *B. sylvarum* | -0.194 | 0.037 | -5.317 | **<0.001** | *** |
